# Remote sensing and foraging data illustrate landscape‐scale considerations for coastal restoration and avian management

**DOI:** 10.1002/eap.70152

**Published:** 2025-12-08

**Authors:** Brock Geary, W. Ryan James, Jordan Karubian, James A. Nelson, Paul L. Leberg

**Affiliations:** ^1^ Department of Biology University of Louisiana at Lafayette Lafayette Louisiana USA; ^2^ Wildlife Futures Program, Department of Pathobiology University of Pennsylvania School of Veterinary Medicine Kennett Square Pennsylvania USA; ^3^ Institute of Environment, Florida International University University Park Florida USA; ^4^ Department of Ecology and Evolutionary Biology Tulane University New Orleans Louisiana USA; ^5^ Department of Marine Sciences University of Georgia Athens Georgia USA

**Keywords:** avian ecology, coastal ecology, habitat selection, movement ecology, remote sensing, restoration ecology, stable isotopes

## Abstract

As coastal restoration projects around the world continue to grow in scale and frequency, it is critical to consider how modified landscapes support wildlife species of concern and broader ecosystem function. In the northern Gulf of Mexico, particularly coastal Louisiana, maintenance of barrier islands serves to protect inland human settlements, and provide critical breeding habitat for many waterbird populations. To remain productive, colonies must also be linked to high‐quality marine foraging areas, though these relationships are rarely evaluated in active restoration areas. To demonstrate how this linkage can be evaluated in dynamic environments at a regional scale, we coupled remote sensing and stable isotope data to generate maps of energetic importance for Gulf menhaden (*Brevoortia patronus*), one of the most ecologically and economically important fish species in the northern Gulf. We then overlaid these maps with foraging movement data from brown pelicans (*Pelecanus occidentalis*) nesting at three of the largest remaining colonies in the state to assess how a novel characterization of their prey distribution matched individual bird movements. We found that the quality of foraging habitat (i.e., menhaden resource quality) had a significant influence on space use decisions of pelicans over space, time, and multiple scales of movement, as well as strong spatial segregation between colonies, highlighting the importance of island placement when considering restoration priorities and wildlife response. Our results show the considerable potential that “*E*‐scapes” hold as a valuable tool for future restoration planning, with utility in assessment of coastal ecosystem function from a spatially explicit, multi‐trophic perspective.

## INTRODUCTION

As global change erodes the ecological and economic value of coastal habitats, these landscapes have naturally been targets of some of the most extensive and costly restoration projects ever proposed (Bayraktarov et al., 2016; Coastal Protection and Restoration Authority of Louisiana, 2023; Reguero et al., 2018). Recovering or retaining ecosystem function alongside these ambitious efforts requires an understanding of the complexities and interdependencies between wildlife and their environments that extend beyond managed areas (Kollmann et al., 2016; Prach et al., 2019). Accordingly, new tools are also needed to achieve these aims in the face of environmental change, as continued post‐restoration research and evaluation of wildlife habitat use are essential to the insurance of sustainable coasts.

In the United States, coastal Louisiana contains nearly half of the nation's wetlands but loses more than 45 km^2^ of this habitat to subsidence, coastal erosion, and sea level rise each year (Bomer et al., 2019). This alarming rate of land loss is among the fastest on Earth and has catalyzed a massive, multi‐stakeholder effort to abate it (Killebrew & Khalil, 2018). Since 2007, over 100 projects have now been implemented following inclusion in the state's Coastal Master Plan, using over 140,000,000 m^3^ of dredged sediment to build or sustain nearly 225 km^2^ of new land, and additional plans are underway to divert the paths of large water bodies, affecting patterns of salinity and sediment deposition over large areas (Coastal Protection and Restoration Authority of Louisiana, 2023). Given that coastal change around the world will be influenced by human manipulation for the foreseeable future (Bayraktarov et al., 2020; Liu et al., 2016; Saunders et al., 2020), it is critical that current restoration efforts throughout the northern Gulf of Mexico adopt more comprehensive, landscape‐scale perspectives to ensure that their resulting inferences and evaluations are informative to future projects worldwide.

There are major gaps in our understanding of how individual restoration actions will function across a larger landscape of concurrent projects to meet the regional needs of wildlife. Many projects employ a strategy of “if you build it, they will come,” which assumes that sites will attract target species and provide natural habitat after a certain period of time (Moore et al., 2020; O'Brien & Arathi, 2021; Suganuma & Durigan, 2022). Although these types of evaluation criteria (e.g., extent of habitat created, degree of use by species of concern) are useful in certain contexts, this approach often fails to capture the contributions of the wider landscape to ecosystem function in restored areas, or how the landscape might benefit from increased wildlife populations in the long term. Improving our understanding of ecological variation among individual project sites in a spatially explicit context will also complement difficult and costly monitoring efforts that follow restoration action, and is necessary to appropriately characterize the scope and quality of benefits gained at regional scales.

Ecosystem function can be increased by improving trophic relationships and energy flow through food webs, but these processes can be challenging to measure over large areas (Gonzalez et al., 2020), especially for species with less specialized diets (James et al., 2022). Novel approaches that couple satellite‐derived environmental data and field‐based tracking of stable compounds in food webs represent an encouraging advance toward a better understanding of resource distributions (Nelson et al., 2020), and the integration of stable isotope‐based trophic information with GIS‐based tools allows the identification of trophically important habitat complexes for individual species at the landscape scale. These energetic resource landscapes or “*E‐*scape” maps have been used to assess patterns of species' distributions and abundance as habitats change over time (James et al., 2022), including responses to distinct events such as vegetative die‐offs (James et al., 2024). In addition to broader ecological insights, these products can also serve as decision support tools in restoration planning, helping to evaluate responses of wildlife to completed restoration actions, or to make predictions regarding future environmental scenarios when impacts to plant communities or physical processes can be estimated.

**FIGURE 1:**
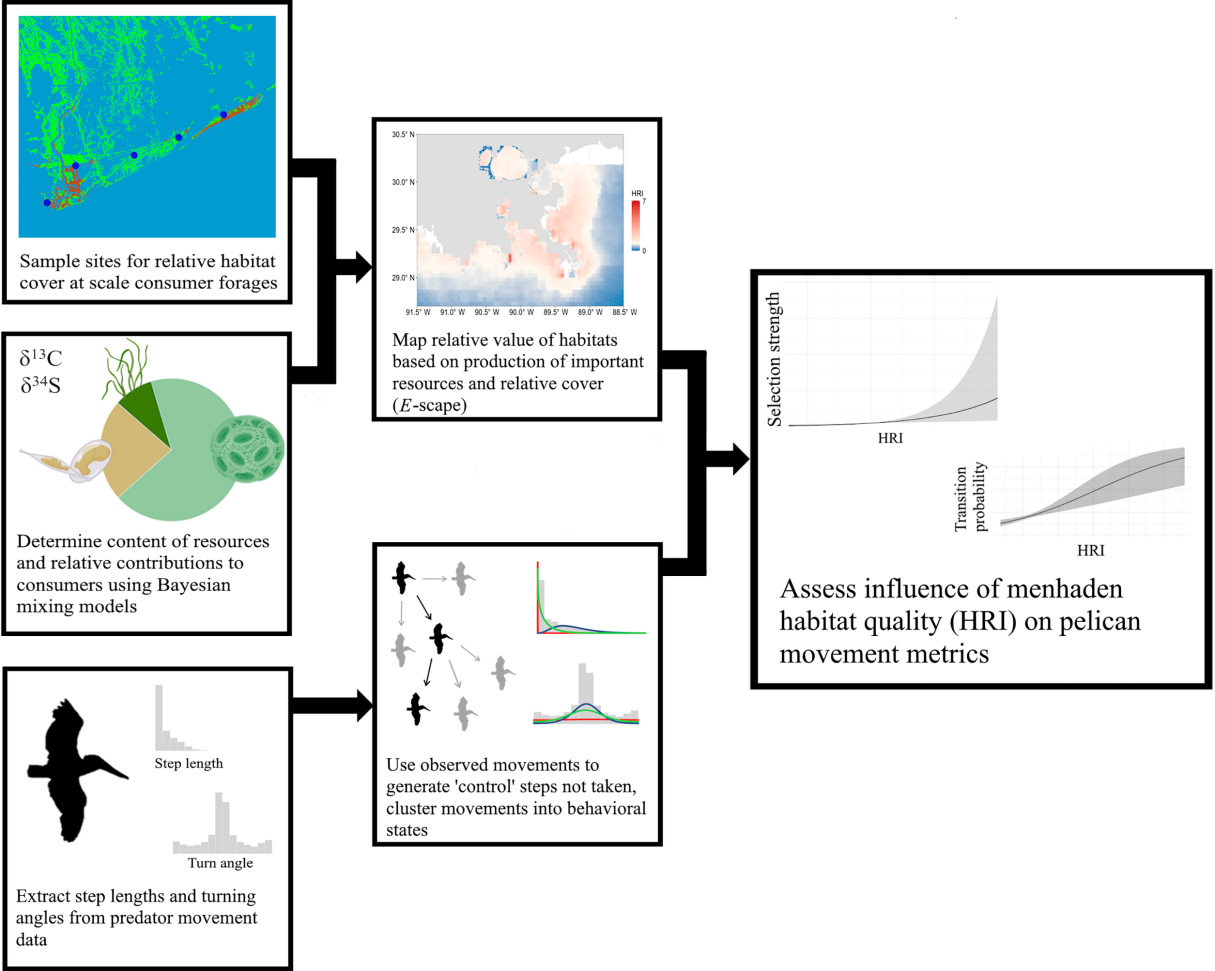
Schematic outlining the methodology used to combine remotely sensed variables, menhaden samples, and brown pelican movement data and assess the ability of *E*‐scapes to predict predator foraging behavior. Created in BioRender (Geary, 2025b; https://BioRender.com/xouvdn5; licensed under CC BY 4.0).

To demonstrate how multi‐trophic, landscape‐level processes can be assessed in the context of an area heavily modified by restoration actions, we developed an *E‐*scape model for Gulf menhaden (*Brevoortia patronus*), one of the most ecologically and economically vital fish species in the northern Gulf of Mexico (Fogarty et al., 1981; Robinson et al., 2015; Vaughan et al., 2007). We then overlaid model outputs with high‐resolution foraging data for a major consumer of menhaden, the brown pelican (*Pelecanus occidentalis*). Many coastal birds are species of conservation concern that also serve as important ecological indicators for the evaluation of large restoration efforts (Ogden et al., 2014a, 2014b). By recording movements of pelicans from three breeding colonies across the Louisiana coast, we illustrate how the *E‐*scape provides a valuable and novel source of information concerning population‐level foraging behavior and potential regional variability in foraging habitat quality across species (Figure 1), all of which carry implications for future restoration decisions and the continued health of the northern Gulf coast.

## METHODS

### E‐scape development

We generated *E‐*scapes for Gulf menhaden, which comprise the majority of brown pelicans' diet in Louisiana (Geary et al., 2020; Shields, 2020). This largely planktivorous species spawns offshore, is born into estuarine nursery habitat, and moves throughout the northern Gulf of Mexico in dense schools that are targeted by diving pelicans. *E*‐scapes assess and map where energetic resources used by a consumer are being produced across the landscape by combining habitat maps and stable isotope analysis (James et al., 2022). Final *E‐*scape maps represent spatial variation of a habitat resource index (HRI) that accounts for both the quantity (amount of each habitat type) and quality (how a consumer is using energetic resources being produced in those habitat types) of trophic function in an area (James et al., 2022).

We determined menhaden basal resource use by conducting stable isotope analysis of menhaden collected near Port Fourchon, Louisiana in August 2016. We collected two aggregates (5 individuals) from both Barataria and Terrebonne Bays, for a total of four aggregate samples (Nelson et al., 2019). We rinsed samples with deionized water, dried them at 50°C for 48 h, ground them whole, and shipped them to the Washington State University Stable Isotope Core Facility for carbon and sulfur stable isotope analysis. We obtained basal resource values (phytoplankton, benthic algae, and marsh grass) as described in Nelson et al. (2019). We ran concentration‐dependent Bayesian mixing models using the “MixSIAR” R package version 3.1.11 (Stock et al., 2018) to determine source contribution and menhaden basal resource use. We used trophic enrichment factors of 1.00 ± 0.63 for carbon and 0.50 ± 0.20 for sulfur. Models ran with three chains for 1,000,000 iterations, with a burn‐in of 500,000 and thin of 500 to allow for adequate model convergence.

We generated menhaden *E*‐scapes using a 10 m resolution coastal Louisiana map from 2013 (Hartley et al., 2017). We used three habitat cover types: “marsh” which was an aggregation of salt, brackish, intermediate, and fresh marsh cover, “water,” and “other,” which contained all non‐marsh and water cover types (James et al., 2021). We used the marsh habitat cover type to indicate the location of marsh production. We calculated edge habitat by measuring the linear distance between the water and marsh habitat cover classes and multiplying by 10 m to generate an area in order to correspond with the location of highest benthic algae production (Appendix S1: Table S1; James et al., 2022). To indicate the location of phytoplankton production, we multiplied the amount of water cover by the relative chlorophyll a concentration of the same area.

We generated two chlorophyll maps to correspond to the two breeding seasons in which we collected pelican movement data (2019 and 2021). We created maps using the 3‐year mean chlorophyll a concentration (2017–2019 and 2019–2021 for 2019 and 2021 pelicans, respectively) from MODIS satellite data during the breeding season (April–August). As we do not possess information on tracked individuals' ages or histories of breeding site fidelity, this is intended to represent the minimum and most relevant (i.e., most recent) amount of potential landscape knowledge that cohorts might possess as they choose nest sites and make broader‐scale foraging decisions, while minimizing the risk of including less relevant information (i.e., when individuals might have nested/foraged elsewhere or not yet been born). We calculated relative chlorophyll concentration by dividing chlorophyll concentration by the maximum chlorophyll concentration for each 3‐year period. We combined habitat cover areas with basal resource use values to calculate an index of energetic importance (IEI) for each of the three resource‐producing habitats with the following formula:

(1) $${\mathrm{IEI}}_{i}=\frac{f_{{\text{source}}_{i}}}{f_{{\text{habitat}}_{i}}},$$

where $f_{{\text{source}}_{i}}$ is the fraction of the contribution of habitat *i* to the total resource use of the consumer based on the results of the mixing model, and $f_{{\text{habitat}}_{i}}$ is the fraction of habitat type *i* that produces source *i* to the overall area at a scale relevant to the movement range of the consumer (i.e., area of a buffer around the sampling point). IEI values are a relative measure of how much a consumer is using a resource relative to the amount that it is available, and IEI values >1 indicate that the consumer is using the resource disproportionately to its availability, while the opposite is true for values <1 (James et al., 2022). We calculated $f_{{\text{habitat}}_{i}}$ using a 500 m radius circular buffer that approximates the scale of foraging behavior of the pelicans. We calculated IEI values for each year of movement data by randomly sampling 1000 points across Louisiana (James et al., 2024; Nelson et al., 2020). We calculated cover areas of marsh and water with the “landscapemetrics” package version 1.5.5 (Hesselbarth et al., 2019), and mean relative chlorophyll concentration with the “exactextractr” package version 0.9.1 (Baston, 2021).

We generated *E*‐scapes for 2019 and 2021 by combining the IEI values and habitat cover within a landscape foraging unit (grid cell with an area that corresponds to the movement range of the consumer; James et al., 2022). We calculated a HRI value within each landscape foraging unit using the following formula:

(2) $${\mathrm{HRI}}_{x}=\sum_{i=1}^{n}{\tilde{\mathrm{IEI}}}_{i}\times f_{{\text{habitat}}_{i,x}},$$

where ${\tilde{\mathrm{IEI}}}_{i}$ is the median of the IEI for the species‐specific source/habitat combination *i* and $f_{{\text{habitat}}_{i,x}}$ is the fraction of habitat *i* to the overall area within landscape foraging unit *x*. HRI is an index that represents a relative measurement of the quality of the habitats for producing the resources used by the consumer based on stable isotope analysis. An HRI value of 1 corresponds to an average level of resource production for the consumer. HRI values >1 mean that the area is better for producing resources (i.e., more energetic resources) being used by the consumer, while HRI values <1 shows that the habitats most important to the production of resources being used by the consumer are underrepresented within the landscape foraging unit (James et al., 2022). We calculated habitat cover areas within each 1 km × 1 km (i.e., at the same scale as 500 m radius buffers) landscape unit as described above.

### Pelican data

We captured brown pelicans nesting on three Louisiana barrier island colonies in the spring of 2019 and 2021. Each colony represents different bay regions in the northern Gulf of Mexico: Raccoon Island in Caillou Bay (29.0519° N, 90.9336° W), Philo Brice Island in Terrebonne Bay (29.1831° N, 90.3462° W), and Queen Bess Island in Barataria Bay (29.3049° N, −89.9576° W). We targeted individuals for capture on similar nests that were concurrently built in black mangrove (*Avicennia germinans*) 1.0–1.5 m in height, and in which chicks of about 5–10 days of age were already being provisioned. We took these measures to control for potential differences among adults in nest site selection or social behavior, which might be further reflected in foraging characteristics. We performed captures, either by hand or handheld net, and attached e‐Obs© tracking units (e‐Obs Digital Telemetry, Gruenwald, Germany) using backpack‐style harnesses made of 0.55″ Teflon ribbon (Bally Ribbon Mills, Pennsylvania, USA) and copper clasps. Tracking units logged GPS locations every 15 min during daylight hours with approximately 5 m accuracy. We visited islands approximately weekly, remotely downloaded tracking data to an e‐Obs handheld base station, and periodically checked nests of tracked individuals to ensure that nests remained active, and that collected data therefore still represented the behavior of provisioning birds.

### Combined analyses

We used the “adehabitatLT” package version 0.3.26 (Calenge, 2015) to identify “complete” foraging trips that included a departure and return to the breeding colony within the same day, and rediscretized them to even 15‐min intervals as recommended for analysis. Using the prepared dataset, we used two different modeling approaches to assess how pelicans responded to foraging conditions as represented by HRI. We first used integrated step selection functions to determine whether HRI values were correlated with individual pelican movements (or “steps”) relative to potential movements at each time point (Avgar et al., 2016; Forester et al. 2009). We generated 50 control points for every observed GPS point in the data, with step lengths drawn from a gamma distribution and turn angles drawn from a von Mises distribution (Avgar et al., 2016). We estimated both parameters according to distributions fit to the data using the “hmmSSF” package version 0.1.0 (Klappstein & Michelot, 2025). We then used a penalized smooth approach to fit a Cox proportional hazards model with the “mgcv” package version 1.9‐1 (Wood, 2017; Wood et al., 2016), which allows nonlinear functions to be incorporated into the model as random effects (Klappstein et al., 2024). We set the event time variable to a constant value of 1, coded weights as a binary argument (grouping observed vs. control points). We included step length, its logarithm, and the cosine of turning angle as fixed effects to characterize and control for pelican movements independently of habitat selection in the model (Avgar et al., 2016). We included a population‐level smooth for the effect of log‐transformed HRI on movements, and a random effect that allowed deviations from the population‐level smooth as varying smooth slopes among individuals (Klappstein et al., 2024). We also included additional random effects for individuals' respective tracking years and breeding colonies as random effects to assess potential regional and/or interannual variation in habitat selection, pooling among groups if their respective term in the model was nonsignificant.

As pelicans in flight do not directly sense the marine conditions that contribute to *E*‐scape construction, we also assessed how menhaden HRI affected broader changes in behavior, again using step lengths and turning angles to construct a three‐state hidden Markov model (HMM) and classify points for visualization using the “moveHMM” package version 1.8 (Michelot et al., 2016) To optimize the starting parameters of the final HMM fit, and simultaneously assess the sensitivity of the model to initial parameter choice, we fit 50 preliminary models that sampled initial parameter values for each behavioral state from uniform distributions of values ranging from 1 to 2000 m for mean/SD step length and 0 to 2 radians of mean/SD turning angle, and consistently recovered the same optimized start values for each state. We included log(HRI) as a covariate in the final model construction to assess potential associations between menhaden habitat quality and changes in pelican behavioral states. All analyses and packages used were implemented in the R software, version 4.4.1 (R Core Team, 2024).

## RESULTS

**FIGURE 2:**
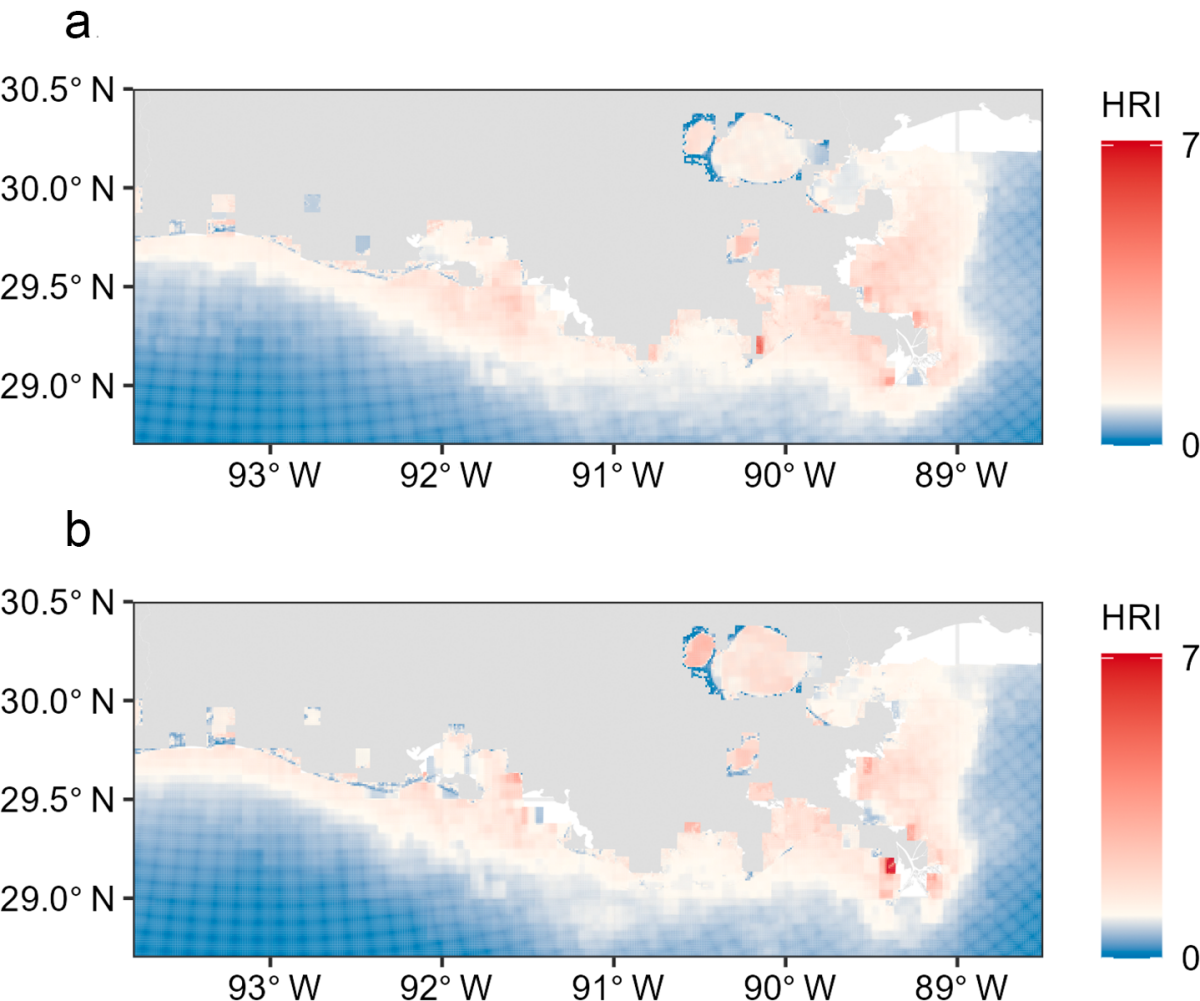
Gulf menhaden (*Brevoortia patronus*) *E*‐scape maps show spatial and temporal variability in habitat resource index (HRI) values. Maps correspond to the landscapes associated with brown pelican (*Pelecanus occidentalis*) GPS data collected in 2019 (a) and 2021 (b).

Of the basal resources detected and analyzed during *E*‐scape construction, phytoplankton had the highest source contribution to menhaden (mean ± SD: 0.68 ± 0.13), followed by benthic microalgae (0.23 ± 0.15) and marsh (0.09 ± 0.07). IEI values suggested high importance of water (median [IQR]: 2019 = 6.2 [4.0–10.8], 2021 = 7.1 [4.6–13.5]) and edge (2019 = 3.9 [2.1–11.0], 2021 = 3.6 [1.6–12.6]) habitat for both years. Marsh had IEI values that were 0.5 (0.2–0.8) in 2019 and 0.3 (0.1–1.0) in 2021. In both years, *E‐*scapes showed higher HRI values near shore that decreased with increasing distance from shore, and spatial heterogeneity in local HRI values around the three colonies (Figure 2).

**FIGURE 3:**
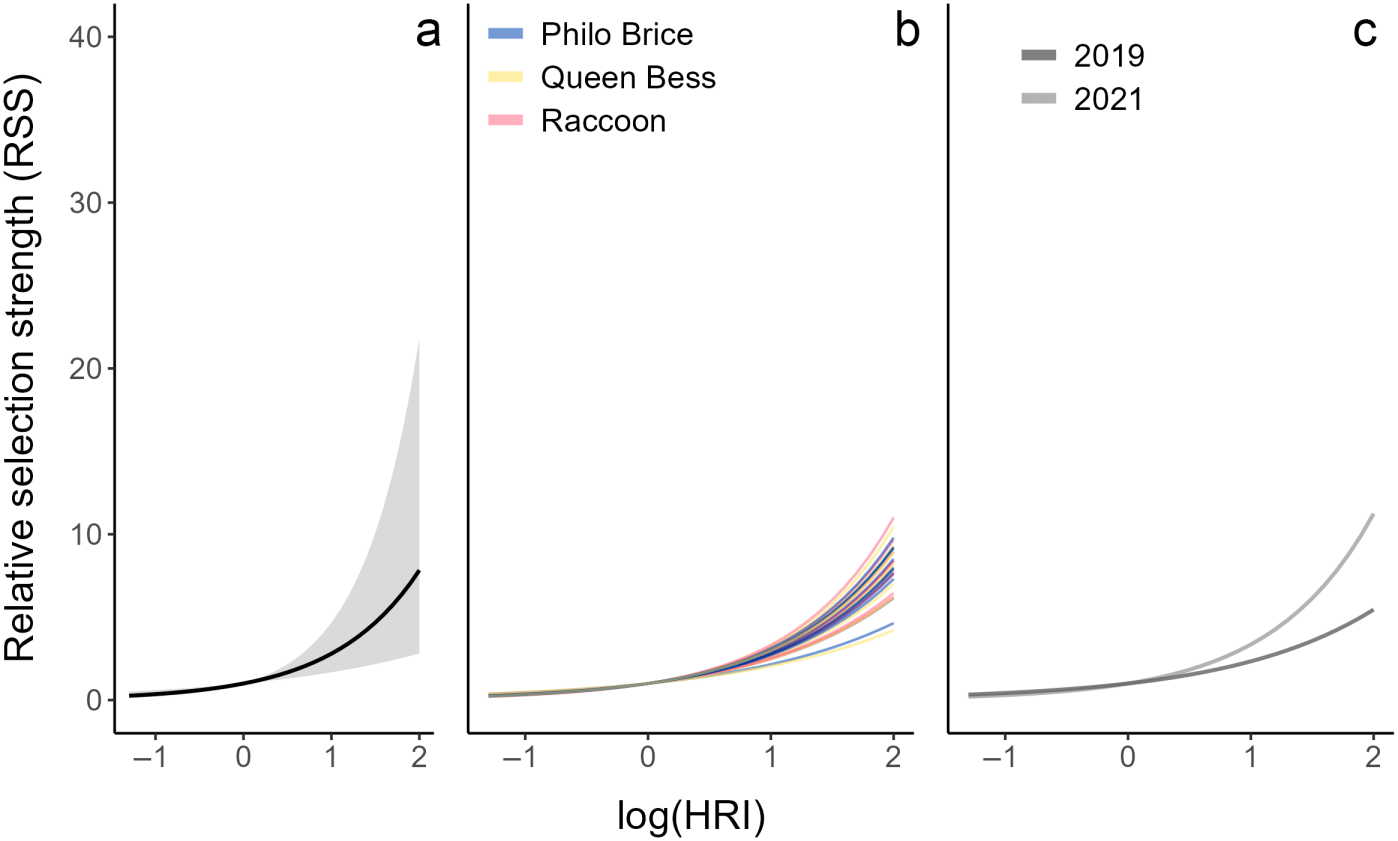
Smooth estimates for the relative selection strength (RSS) of log‐transformed habitat resource index (log(HRI)) values influencing brown pelican (*Pelecanus occidentalis*) foraging movements. (a) shows the population‐level smooth across all tracked individuals (shaded ribbon represents 95% CI); (b) shows smooths corresponding to individual birds, with lines colored according to each bird's nesting colony (illustrated here to show lack of pattern; colony effect not included in this model); (c) shows variation in slope between the two tracking years.

After initial processing, a total of 7897 locations from 28 birds generated the steps and turns used in the analysis. Fitting of the full step selection model resulted in a non‐significant effect of breeding colony on the random smooths (χ^2^ = 0.01, EDF = 0.03, RDF = 2, *p* = 0.66), and it was removed from the final model, leaving variation around the population‐level smooth among individuals within years. We found a significant positive association with HRI (β = 1.05 ± 0.33, *z* = 3.13, *p* = 0.002), with a nearly three‐fold increase in the likelihood of a “typical” pelican taking a step per one‐unit increase in log(HRI) (Figure 3). We also found variation among individuals (χ^2^ = 12.15, EDF = 5.44, RDF = 27, *p* = 0.02) and years (χ^2^ = 9.26, EDF = 0.80, RDF = 1, *p* = 0.03), but all individuals showed a similar positive response to energetically important menhaden habitat.

**FIGURE 4:**
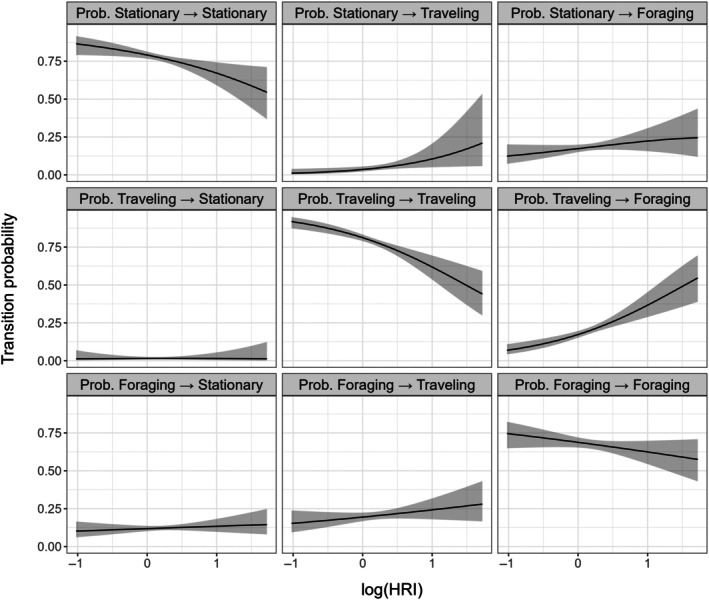
Probabilities of transitions between behavioral states by brown pelicans (*Pelecanus occidentalis*) as a function of *E*‐scape habitat resource index (HRI) values, as derived from a hidden Markov model. Shaded ribbons represent 95% CIs.

A three‐state model of pelican movement distinguished apparent stationary (step length: 5.41 ± 5.05 m, turn angle: μ = 0.42, κ = 0.07), traveling (step length: 5203 ± 2903 m, turn angle: μ = 0.03 rad, κ = 1.62), and foraging (step length: 1176 ± 1353 m, turn angle: μ = −0.05 rad, κ = 0.89) behaviors (Appendix S1: Figures S1 and S2). Stationary behaviors mostly occurred on or near the colony, while off‐colony movements generally consisted of long‐distance traveling bouts interspersed with foraging efforts. We found that state transitions between behaviors were broadly correlated with *E‐*scape HRI values; higher HRI values were negatively correlated with the probability of a bird remaining in any given state, and positively correlated with the tendency to switch to “foraging” from either other state, though only the 95% CI for the “traveling ➔ foraging” transition did not include zero (from stationary β = 0.41 (CI: −0.04 to 0.88); from traveling β = 1.02 (0.62–1.42); Figure 4).

**FIGURE 5:**
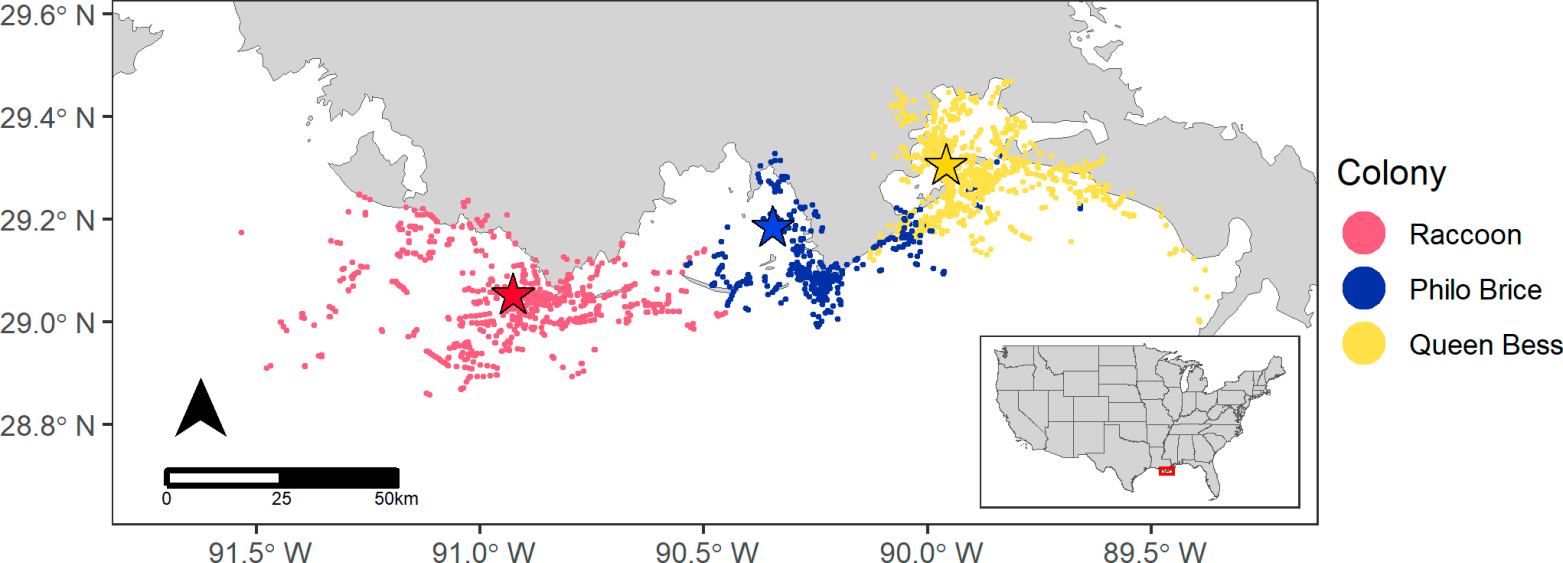
Distribution of brown pelican (*Pelecanus occidentalis*) foraging points (as identified by a Hidden Markov model) demonstrating spatial segregation of foraging grounds among the three nesting colonies where birds were captured for GPS telemetry. Stars represent breeding colony locations, while circles represent locations of foraging pelicans.

Using the Viterbi algorithm to classify each segment into one of three states according to the model (Zucchini & MacDonald, 2009), we also observed substantial segregation in areas occupied while foraging between birds from different colonies, including avoidance of some areas near known smaller colonies not included in this study. We saw almost no overlap among birds from different colonies in the portions of the northern Gulf utilized for foraging, apart from some overlap in the vicinity of Grand Isle/Grand Terre by birds from Philo Brice and Queen Bess Islands (Figure 5).

## DISCUSSION

Our results demonstrate that *E‐*scapes can provide spatially explicit inferences to help restore landscapes to high levels of ecosystem function and maximize future benefits to wildlife. By integrating data from field measurements to satellite observations, from isotopes to top predators, we illustrate a valuable new approach to better anticipate how the placement of new habitats on a landscape may affect species that occupy them, improving the efficacy of complex and costly restoration efforts. Based on inferences from a menhaden *E‐*scape, actions that affect marine areas can be assessed with regard to their potential ecological effects on menhaden populations, established pelican colonies, or proposed island restoration targets. Similarly, multispecies approaches incorporating two or more *E*‐scapes could aid prioritization of activities within projects, improving benefits to breeding birds based on predicted variation in habitat use and foraging performance. At a regional scale, these considerations can contribute to the support of diverse and productive ecosystems as land‐building goals are achieved.

*E‐*scapes can also serve as spatial models by which predictions can be generated to address broader ecological questions at multiple scales. For example, we found evidence of varying degrees of selection for more energetically important menhaden habitats between the two tracking years, but also among birds within years, suggesting that individual foraging ability may interact with annual variability in marine conditions to shape population‐level foraging (and breeding) performance (Geary et al., 2019). As shown previously, brown pelicans did not occupy the highest quality areas for menhaden throughout the breeding season, as menhaden also use nearshore habitats where it is generally difficult for pelicans to dive (Geary et al., 2020). While not a surprising result in this case, it nonetheless provides an example of how system knowledge can guide inferences based on what might be predicted purely by *E‐*scape output, or potentially guide subsequent research questions (e.g., investigating avoidance of apparently high‐quality offshore habitat).

Spatial segregation of the three pelican colonies' respective foraging areas suggests that coastal birds may also experience variable impacts as individual islands are restored across the landscape. The movements of nesting birds are limited by many factors, such as the influence of social cues during foraging, (Thiebault et al., 2014), competition, and the need to regularly return and provision their young. Poor marine conditions in the vicinity of restored sites may limit reproductive success and potential reuse of colonies (Naves et al., 2006), and an ecological trap may result when other sites are not available and competition is exacerbated within a colony (Kristan, 2003; Naves et al., 2006). In the case of Louisiana's brown pelicans, this is a scenario of real concern, as current breeding efforts are largely concentrated at only a few sites, despite additional islands being managed for their use over the last 20 years (Selman et al., 2016; Walter et al., 2013). If increasing bird densities affect foraging effort and nesting success, the selection of future restored colonies could also be impacted, and pairing the *E‐*scape approach with relevant survey or harvest data could provide valuable insights that help guide future management priorities. As future coastal projects are advanced that modify marine conditions and resource distributions, it will be critical to maintain focus on ecological impacts that may arise for wildlife moving between marine and terrestrial habitats.

Both regulatory and management actions in restored systems could have large effects on the distribution of important resources in our study area and elsewhere. For example, recent actions and proposals in the northern Gulf have included the creation of a major sediment diversion, construction of multiple offshore wind farms, and implementation of new menhaden harvest regulations. The Mid‐Barataria sediment diversion, if implemented, was projected to increase marsh habitat while also impacting salinity, nutrients, water clarity, and ultimately forage fish abundance and distributions within Barataria Bay (de Mutsert et al., 2017; US Army Corps of Engineers, 2022). Offshore wind farms may increase bird mortality and affect movement patterns (Peschko et al., 2020; van Bemmelen et al., 2024), although negative effects are not consistently observed across species (Lindeboom et al., 2011). A recent regulatory change pushing menhaden harvests further away from Louisiana's shorelines (Louisiana Administrative Code title 76 § VII‐307) could also affect the distribution and availability of this important foraging resource to pelicans and other marine wildlife. Improved understanding of the distribution of foraging resources, combined with bird responses to them, could help assess the effects of harvest regulations, energy developments and restoration actions, and help direct activities where they might minimize wildlife conflicts and food web disruption.

While our results span multiple bay regions, and the colonies monitored represent a substantial proportion of pelican breeding activity in Louisiana, contextualization of our study would be improved with additional data collection over space and time. This is not necessarily the case in all respects; while our isotopic inferences are constrained to the sampled places and times of the study, and sample sizes for menhaden are limited, previous studies have shown narrow ranges for isotope values in adult menhaden in the northern Gulf of Mexico, and agree with the values we present (Olsen et al., 2014). However, additional bird tracking on the smaller colonies found in the region could more thoroughly describe foraging overlap among breeding populations, and future monitoring would provide information on longer‐term changes in habitat quality for menhaden and pelicans. While there are difficulties associated with such efforts at broader scales, such coordination may be necessary to fully appreciate baseline fluctuations in habitats and behaviors, and to accurately assess deviations that occur in response to human intervention.

Consideration of the energetic perspective when evaluating coastal restoration efforts highlights the many interconnected processes that may be underappreciated when assessing areal habitat creation or avoiding clear harm to wildlife. Changes to habitat quality for Gulf menhaden would have cascading effects on estuarine and marine food webs, and a lack of menhaden control on plankton populations could exacerbate problems related to marine hypoxia, which has persisted in the region for decades (Langseth et al., 2014; Rabalais et al., 2002; Short et al., 2021). Failure to establish healthy seabird colonies on restored barrier islands may also impact island integrity, as these species contribute nutrients to these terrestrial environments and often improve vegetation growth and substrate stability (Grant et al., 2022). Our approach here demonstrates how novel tools can integrate a data‐driven energetic perspective within the decision‐making process of large restoration programs. Without maintaining these functions, restoration efforts may fail in the long term or require extensive maintenance, wasting resources and potentially diverting them from other projects.

Strains on funding and physical material are likely to carry societal implications, as degraded coastal ecosystems will fail to protect regions relied upon for residences, industries, and culture. Beyond direct impacts to human welfare, opinions toward future restoration can also be negatively affected, ultimately jeopardizing the future of coastal management efforts. We advocate for more widespread use of this approach in systems where focal species (either indicators or species of concern) are prioritized in the definition and evaluation of restoration outcomes (e.g., habitat creation, stable/increasing populations), from which broader monitoring programs can be built and implemented.

## CONFLICT OF INTEREST STATEMENT

The authors declare no conflicts of interest.

## ETHICS STATEMENT

We conducted all handling of birds per protocols of the University of Louisiana at Lafayette's Institutional Care and Use Committee (IACUC 2017‐8717‐038‐FLDS), as well as Louisiana Department of Wildlife and Fisheries Scientific Collecting Permits (WDP‐19‐020 and WDP‐21‐018).

## Supporting information


Appendix S1.

## Data Availability

Data and code (Geary, 2025a) are available in Zenodo at https://doi.org/10.5281/zenodo.17195479.
