# Supplementary material for: Remote sensing and foraging data illustrate landscape‐scale considerations for coastal restoration and avian management
Source: Ecol Appl. 2025 Dec 8;35(8):e70152. doi: 10.1002/eap.70152 (PMC12683706; doi:10.1002/eap.70152)
Supplement: Supplementary file 1 — Appendix S1. [file EAP-35-e70152-s001.pdf]

Appendix S1

Remote sensing and foraging data illustrate landscape-scale considerations for coastal restoration and avian management

Brock Geary, W. Ryan James, Jordan Karubian, James A. Nelson and Paul L. Leberg

Ecological Applications

Table S1. Isotope ratios of aggregate Gulf menhaden (*Brevoortia patronus*) samples, collected to determine basal resource use for *E*-scape mapping.

Sample	$\delta^{13}C$	$\delta^{15}N$	$\delta^{34}S$
Barataria Bay aggregate #1	-22.18	9.27	9.50
Barataria Bay aggregate #2	-20.25	12.26	15.48
Terrebonne Bay aggregate #1	-20.69	8.23	8.81
Terrebonne Bay aggregate #2	-21.08	9.14	11.19

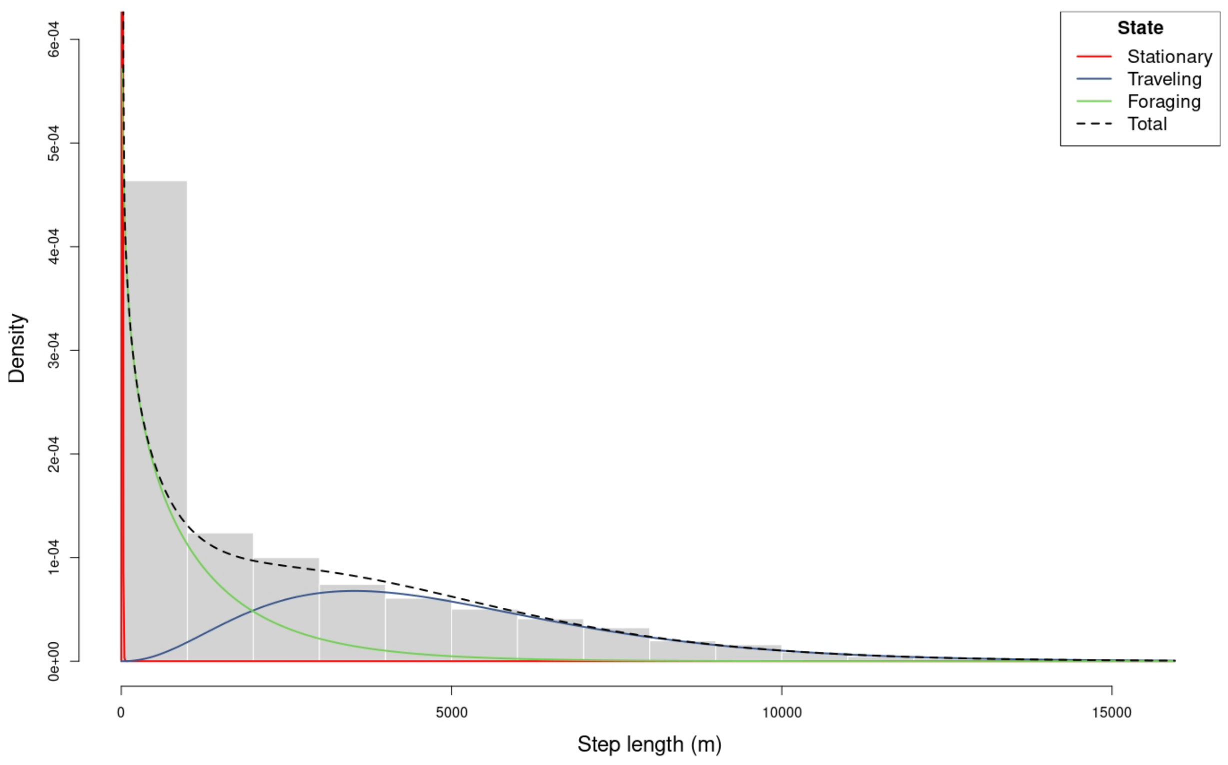

Figure S1. Fitted state-dependent distributions of step lengths between relocations from a Hidden Markov model of brown pelican (*Pelecanus occidentalis*) GPS locations. A histogram represents the full data set.

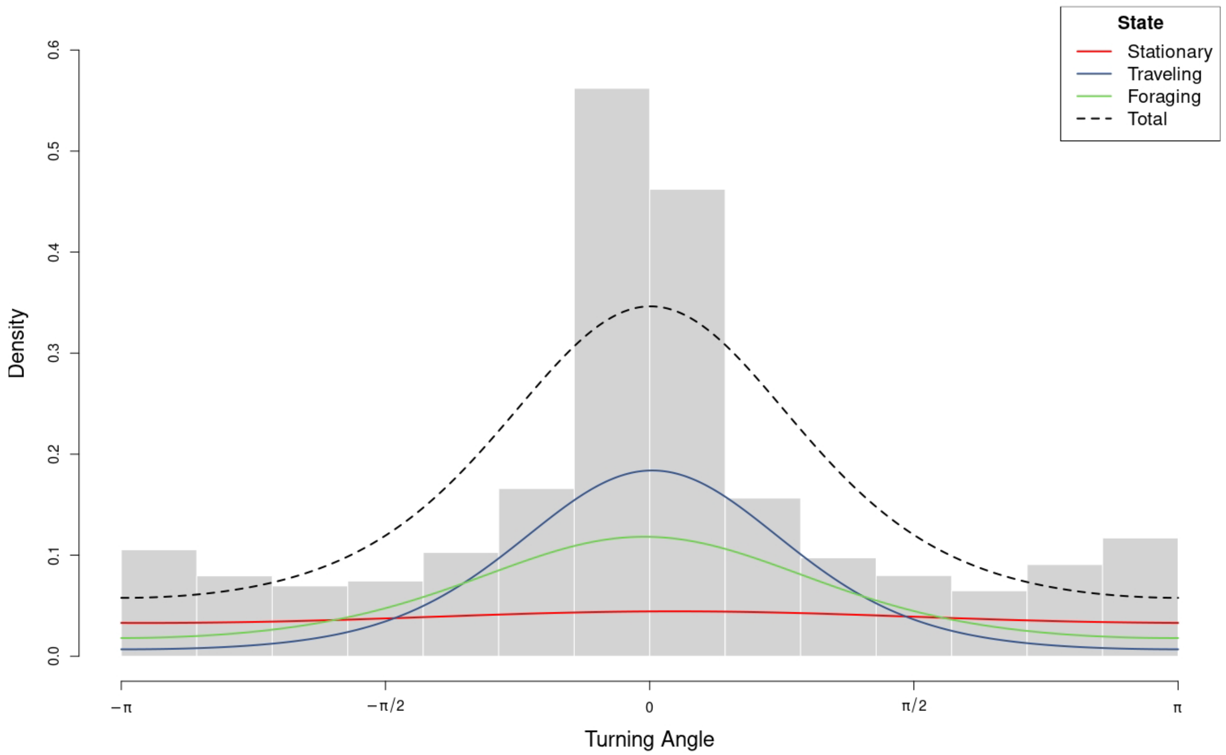

Figure S2. Fitted state-dependent distributions of turning angles between relocations from a Hidden Markov model of brown pelican (*Pelecanus occidentalis*) GPS locations. A histogram represents the full data set.
